# Omics Approaches Applied to *Penicillium chrysogenum* and Penicillin Production: Revealing the Secrets of Improved Productivity

**DOI:** 10.3390/genes11060712

**Published:** 2020-06-26

**Authors:** Carlos García-Estrada, Juan F. Martín, Laura Cueto, Carlos Barreiro

**Affiliations:** 1 INBIOTEC (Instituto de Biotecnología de León). Avda. Real 1—Parque Científico de León, 24006 León, Spain; laura.cueto@inbiotec.com (L.C.); carlos.barreiro@inbiotec.com (C.B.); 2 Departamento de Ciencias Biomédicas, Universidad de León, Campus de Vegazana s/n, 24071 León, Spain; 3 Área de Microbiología, Departamento de Biología Molecular, Facultad de Ciencias Biológicas y Ambientales, Universidad de León, 24071 León, Spain; jf.martin@unileon.es; 4 Departamento de Biología Molecular, Universidad de León, Campus de Ponferrada, Avda. Astorga s/n, 24401 Ponferrada, Spain

**Keywords:** penicillin, *Penicillium chrysogenum*, omics, beta-lactam antibiotics

## Abstract

Penicillin biosynthesis by *Penicillium chrysogenum* is one of the best-characterized biological processes from the genetic, molecular, biochemical, and subcellular points of view. Several omics studies have been carried out in this filamentous fungus during the last decade, which have contributed to gathering a deep knowledge about the molecular mechanisms underlying improved productivity in industrial strains. The information provided by these studies is extremely useful for enhancing the production of penicillin or other bioactive secondary metabolites by means of Biotechnology or Synthetic Biology.

## 1. Introduction

There are few examples of industrial microbial processes as deeply characterized as penicillin production by the filamentous fungus *Penicillium chrysogenum*. In the timeframe of more than 90 years since the discovery of penicillin by Sir Alexander Fleming, scientists have successfully deciphered the main mechanisms behind penicillin biosynthesis and improved productivity in industrial strains. Moreover, recent phylogenetic studies have concluded that Fleming’s isolate (*Penicillium notatum*), the parental strain *P chrysogenum* NRRL-1951 and the full genome sequenced strain *P. chrysogenum* Wisconsin 54-1255 are *Penicillium rubens* [1]. However, a vast literature including the classic names of these microorganisms is available and, therefore, that nomenclature is still used for the sake of clarity.

From the historical point of view, the discovery of penicillin and the development of industrial processes aimed at the massive overproduction of this antibiotic, represent one of the most important milestones in human medicine and conform an interesting story about serendipities, Second World War, Nobel prizes, industrial secrets, and above all, the huge unprecedented collaborative effort made between scientists from industry and academia [2,3,4]. The interest shown by the pharmaceutical industry in penicillin in the middle of the 20th century pushed forward the research activities, not only in the technology for the massive production of this metabolite (media composition, fermentation parameters, etc.), but also in the mold that naturally produces it. Therefore, different companies applied strain improvement programs to ancestor lines of *P. chrysogenum* through classical mutagenesis and selection, thus giving rise to the current industrial strains. These strains can produce more than 50 mg/mL (83,300 i.u./mL), which represents product titers and productivities of at least three orders of magnitude above those provided by the ancestor strains [5,6].

**Figure 1:**
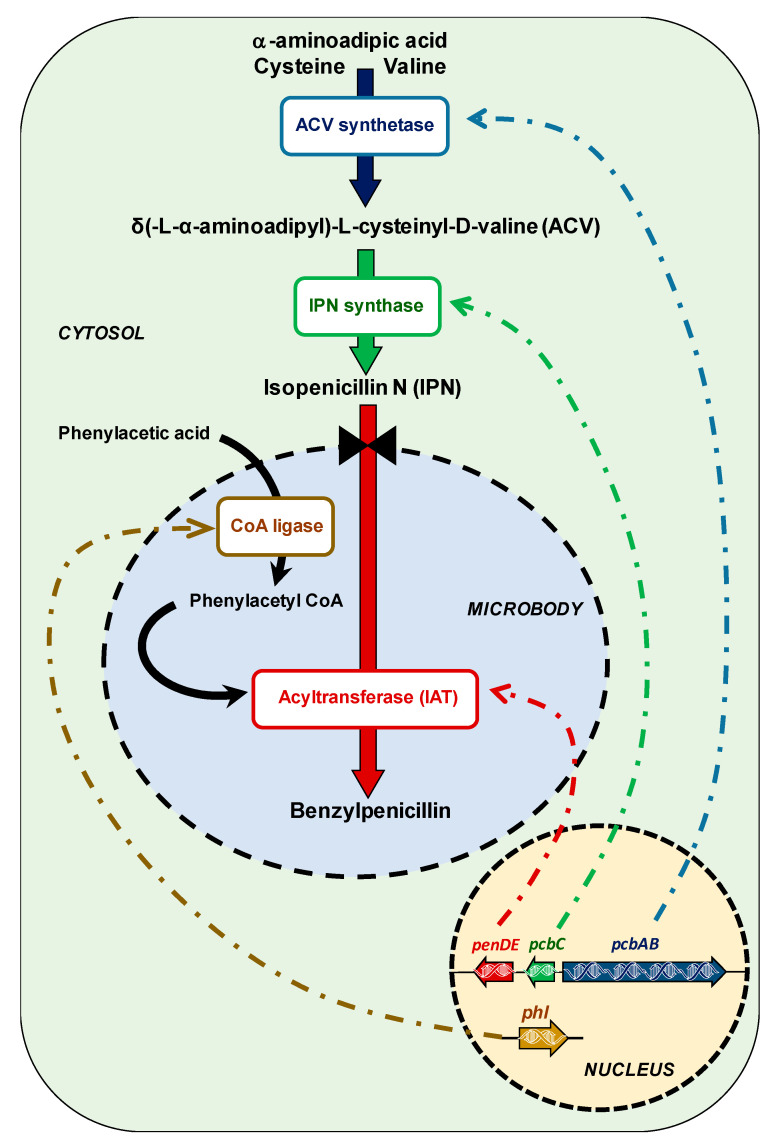
Schematic representation of the benzylpenicillin biosynthetic pathway.

The advances in biochemistry, molecular biology, and genetics have allowed the biochemical and genetic characterization of the penicillin biosynthetic pathway, which is compartmentalized between cytosol and microbodies [4,7] (Figure 1).

**Figure 2:**
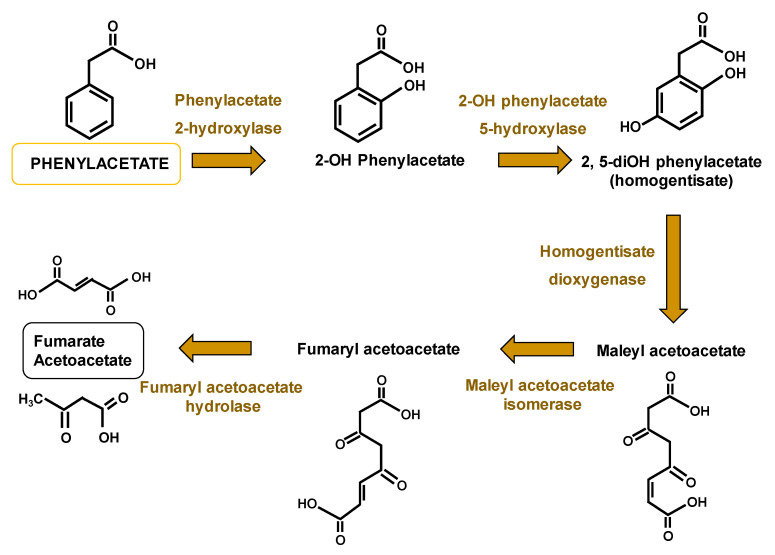
Catabolism of phenylacetic acid in *P. chrysogenum* via the homogentisate pathway.

Briefly, the molecule of penicillin is synthesized from the precursor amino acids α-aminoadipic acid, L-cysteine, and L-valine. They are non-ribosomally condensed by the large multifunctional protein L-δ(α-aminoadipyl)-L-cysteinyl-D-valine (ACV) synthetase (ACVS) (encoded by the 11-kbp *pcbAB* gene), which requires activation by the addition of the CoA-derived 4′-phosphopantetheine arm in a reaction catalyzed by the ancillary protein phosphopantetheinyl transferase. Then, ACV is cyclized by the isopenicillin N (IPN) synthase or cyclase, which is encoded by the *pcbC* gene. After cyclization, the bicyclic structure (penam nucleus) of IPN is formed in the cytosol. Finally, IPN enters the microbody matrix, where the heterodimeric protein acyl-CoA: IPN acyltransferase (IAT) (encoded by the *penDE* gene) replaces the α-aminoadipyl side-chain of IPN with a hydrophobic side-chain, which must be previously activated as thioester with CoA by aryl-CoA ligases in microbodies. The side-chain precursor in the specific case of benzylpenicillin is phenylacetic acid, which is activated in the form of phenylacetyl-CoA. Phenylacetic acid has been extensively used as the benzylpenicillin side-chain precursor. This weak acid is metabolized in *P. chrysogenum* through at least two ways; incorporation of the benzylpenicillin molecule or catabolism via the homogentisate pathway (Figure 2) [8,9,10].

Regulation and control of penicillin biosynthesis and production have been subjected to a deep study to optimize penicillin titers. It is well-known that penicillin production takes place, preferentially, under stress nutrient and low growth rate conditions. Carbon, nitrogen, or phosphorous availability, together with other factors (pH, aeration, certain amino acids, or media composition) have a strong influence on the production process. Penicillin biosynthesis is regulated by complex mechanisms controlled by different regulators. Some examples of either specific or global transcription factors include CreA, PacC, Nre, PcRFX1, PcFKH1, LaeA, and the Velvet complex [11,12,13]. CreA (Cre1) mediates carbon catabolite repression in fungi [14], and is an ortholog of the *S. cerevisiae* factor (MIG1) responsible for repression of glucose-regulated genes [15]. Glucose and other sugars have a negative effect on penicillin biosynthesis in *P. chrysogenum*. Glucose dramatically reduces the transcription of the three penicillin biosynthetic genes [16], CreA-1 being the main cis-acting element regulating carbon repression of the *pcbAB* gene [17]. PacC is a transcriptional activator that regulates pH-dependent gene expression is filamentous fungi [18]. Biosynthesis of penicillin by *P. chrysogenum* is favored under alkaline conditions, and PacC seems to regulate the expression of the penicillin biosynthetic genes, since several putative binding sites for this transcription factor are present in the promoter regions of these genes [19]. The transcription factor AreA (Nre in *P. chrysogenum*) is able to activate, through a de-repression mechanism, those genes involved in the use of alternative nitrogen sources [20]. It is well-known that ammonium decreases penicillin biosynthesis in *P. chrysogenum* [21]. Nre-mediated nitrogen repression of the expression of the penicillin biosynthetic genes is likely, since interaction of this transcription factor with the *pcbAB-pcbC* promoter region has been reported [22]. The global regulator PcRFX1 (ortholog of the *Acremonium chrysogenum* CPCR1 [23]) is a winged helix transcription factor that controls penicillin biosynthesis by binding the promoter region of the biosynthetic genes [24]. Another subclass of winged helix transcription factor is represented by PcFKH1, a member of the forkhead family of regulators. The first forkhead regulator characterized in filamentous fungi was the *A. chrysogenum* AcFKH1, which was reported to act in association with CPCR1 [25]. In *P. chrysogenum*, PcFKH1 exerts a positive control in the expression of the *penDE* gene and ancillary genes of the penicillin biosynthesis, such as *phlA* (encoding phenylacetyl-CoA ligase) and *ppt* (encoding phosphopantetheinyl transferase), and regulates conidiation and spore pigmentation [26]. LaeA is a nuclear methyltransferase that acts as a global secondary metabolite regulator. It was described for the first time in *Aspergillus* species with a putative role in chromatin remodeling [27]. In *P. chrysogenum*, PclaeA not only controls penicillin biosynthesis, but also pigmentation and asexual differentiation [28]. LaeA interacts with members of the Velvet Complex, which comprises several proteins, such as VeA (VelA), VelB, VelC, and VosA involved in coordinating secondary metabolism and differentiation processes [29,30]. In has been observed that in *P. chrysogenum* LaeA, VeA, and VelC behave as positive regulators of the penicillin biosynthesis, whereas VelB has the opposite effect, and that PcVelB and PcVosA promote conidiation, while PcVelC has an inhibitory effect [31,32].

Due to the interest of *P. chrysogenum*, most of the early research in this microorganism was focused on the characterization of the modifications introduced by industrial strain improvement programs. Hence, it was reported that the genomic region including the penicillin gene cluster is amplified in tandem repeats in many of the improved penicillin-producing strains [33]. It was also observed that catabolism of phenylacetic acid via the homogentisate pathway (Figure 2) was diminished in Wisconsin 54–1255, and presumably, in derived strains as well, which leads to reduced degradation of the side-chain precursor and penicillin overproduction [34,35]. More recently, with the advances in confocal microscopy, it was possible to conclude that microbodies (peroxisomes), the organelle where benzylpenicillin is formed from IPN and phenylacetyl-CoA, are more abundant in high-producer strains [36].

The arrival of the omics era with the publication of the *P. chrysogenum* Wisconsin 54-1255 genome [36] paved the way for genomics, transcriptomics, proteomics and metabolomics studies focused on a deeper characterization of global modifications occurring in industrial strains under production conditions. The main omics studies carried out so far in different strains of *P. chrysogenum* are reviewed in this article. Integration of the knowledge obtained from these techniques has contributed to unveiling the secrets behind penicillin production, thus contributing to the full exploitation of *P. chrysogenum* as a microbial factory.

## 2. Genomics

The first *P. chrysogenum* genome sequenced was that of the international reference strain *P. chrysogenum* Wisconsin 54-1255 [36]. Some differences between *P. notatum* and *P. chrysogenum* strains have been reported. These include mutations in the genes encoding the phenylacetic acid-2 hydroxylase, *pahA*, that decreases drastically the degradation of phenylacetic acid in *P. chrysogenum* NRRL1951 as compared to *P. notatum* [34,35]. Maintenance of high intraperoxisomal levels of phenylacetic acid and at the same time reducing its cytoplasmic concentration to avoid its toxicity is an important parameter in the production of benzylpenicillin [37]. In addition, *P. notatum* and *P. chrysogenum* differ greatly in the transcription ability of the penicillin biosynthetic genes in response to the PTA1 transcriptional activator [38]. Differences cannot be extended to other genes since the *P. notatum* genome has not been published yet.

### 2.1. The P. chrysogenum Wisconsin 54-1255 Genome

The genome of the *P. chrysogenum* Wisconsin 54-1255 reference strain has 32.19 Mb, distributed in 49 supercontigs, and encodes 12,943 predicted proteins [36]. The mutations originated in this genome during the improvement strain program are not traceable since the complete genome sequence of original wild-type strain *P. chrysogenum* NRRL 1951 is not available [39]. However, some DNA sequences of *P. chrysogenum* NRRL 1951 were obtained and compared with the prototype genome sequence of *P. chrysogenum* Wisconsin 54-1255 [40] and several mutations produced during the early steps of the classical strain improvement programs have been detected. There is not only an increase of penicillin production from *P. chrysogenum* NRRL 1951 to *P. chrysogenum* Wisconsin 54-1255, but also a partial or complete silencing of several polyketide and non-ribosomal peptide gene clusters. The only non-ribosomal peptide synthetase (NRPS) gene that accumulated a point mutation is PssA (Pc16g03850), which encodes an enzyme involved in coprogen (a siderophore involved in chelation and transport of iron) biosynthesis. The improved strains still show coprogen synthetase activity, which indicates that apparently, this mutation did not inactivate the enzyme [40]. Genes encoding several polyketide synthase (PKS) enzymes were altered in the strain improvement programs. Two SNPs occurred in the gene encoding PKS2 (Pc13g04470), which shows similarity to a diketide synthase LovF involved in lovastatin biosynthesis, likely inactivating this enzyme. Mutations were also introduced in the PKS7 (Pc16g11480) and PKS8 (Pc21g00960) encoding genes, which encode two different uncharacterized enzymes. Other mutated polyketide synthetases encoding genes were PKS14 (Pc21g12440) and PKS15 (Pc21g12450), which belong to a large secondary metabolite gene cluster. The PKS17 (Pc21g16000) gene, encoding a protein with similarity to naphthopyrone synthases (involved in the biosynthesis of a precursor for the formation of a conidial pigment), has three nucleotide substitutions with unclear effect on pigment formation. The gene cluster of PKS18 (Pc22g08170), likely a 6-methylsalicylic acid synthase (6-MSA) involved in the synthesis of secondary metabolites derived from 6-MSA, includes a putative MFS transporter (Pc22g08250) with a single mutation. Finally, other mutated PKS encoding genes, PKS12 (Pc21g05070) and PKS13 (Pc21g05080), which are expressed in opposed orientation, are potentially involved in the biosynthesis of sorbicillinoid pigments. These mutations correlate with the decrease yellow pigmentation due to sorbicillinoids in the high penicillin-producing strain as compared to the parental strain *P. chrysogenum* NRRL 1951 [40].

In summary, most of these mutations seem to have reduced or suppressed the formation of polyketides or non-ribosomal peptides, thus saving precursors for penicillin biosynthesis.

### 2.2. The P. chrysogenum P2niaD18 Genome

The *P. chrysogenum* P2niaD18 strain, containing a *niaD* mutation [41] for a nitrate reductase, derives from the PanLab strain P2 [42]. Specht and co-workers [43] sequenced the genome of this strain and found that there is a duplication of the penicillin gene cluster (pen) in chromosome I of *P. chrysogenum* P2niaD18. In addition, they found two DNA fragment translocations, one between chromosomes II and III that probably originated the *niaD* mutation, and other between chromosomes III and IV. A total of 11,749 open reading frames (ORF´s) were found in the *P. chrysogenum* P2niaD18 genome most of which were identical or near identical to those of *P. chrysogenum* Wisconsin 54-1255.

### 2.3. Industrial Strains Genomes

Industrial penicillin high-producing strains have been developed in several pharmaceutical companies. Several publications focusing on the amplified regions (containing the *pen* gene cluster) of the penicillin high-producing strains *P. chrysogenum* ASP-78 and *P. chrysogenum* E1 provided by Antibióticos SA in León (Spain), and the *P. chrysogenum* BW 1890 series of strains of Smith Kline Beecham (SKB, United Kingdom) have been published [33,44]. Three additional industrial laboratories provided the complete genome sequences of strains *P. chrysogenum* P2niaD18 [43], *P. chrysogenum* DS17690 [40] and *P. chrysogenum* NCPC10086 [45].

The DNA region that encodes the *pen* gene cluster and adjacent sequences belongs to an amplified region of 106,5 kb in strain *P. chrysogenum* ASP-78 and 57.9 kb in *P. chrysogenum* E1. This region is amplified head to tail in tandem repeats and consists of 5–6 copies in *P. chrysogenum* ASP-78 and 12–14 copies in *P. chrysogenum* E1 [33,46] linked by a conserved hexanucleotide sequence TTTACA. In the SKB series of improved strains the amplification ranged from one copy (strain BW1900A) to 50 copies (strain BW1952) [44].

### 2.4. Analysis of the Mutations in the Genome of Penicillin High-Producing Strains

Comparison of the industrial strains *P. chrysogenum* DS17690 and *P. chrysogenum* NCPC10086 genomes with that of *P. chrysogenum* Wisconsin 54-1255 showed that many mutations occur in the *P. chrysogenum* industrial strains. The first difference affects the number of copies of the *pen* cluster that is present in eight copies in *P. chrysogenum* DS17690 [40] as compared to one copy in the *P. chrysogenum* Wisconsin 54-1255 strain. The improvement program for penicillin production resulted in accumulated mutations in 11 genes for NRPSs and 20 genes for PKSs. Also, the regulatory *laeA* and *velA* genes of *P. chrysogenum* suffered mutations as analyzed by Veiga et al. and Martín [47,48], which could affect the expression of secondary metabolites [28,40,47].

The genome of *P. chrysogenum* NCPC10086, an industrial strain developed by the North China Pharmaceutical Group Corporation was published by Wang et al. [45]. This strain was phylogenetically reported to be close to *P. chrysogenum* Wisconsin 54-1255. The size of *P. chrysogenum* NCPC10086 genome was 32.3 Mb with a GC content of 48.9%, similar to that of *P. chrysogenum* Wisconsin 54-1255. By using improved sequencing and bioinformatic tools these authors identified in *P. chrysogenum* NCPC10086 13,290 ORFs, most of them identical or close to 100% identical in both strains. Sixty-nine new genes, not found in *P. chrysogenum* Wisconsin 54-1255 genome, were reported [45]. Some of these genes may correspond to redefined nucleotide sequences as compared to the genome of *P. chrysogenum* Wisconsin 54-1255. These new genes are involved in amino sugar metabolism, nucleotide metabolism, glycogen metabolism, and oxidative phosphorylation that may result in improved energy availability. Of special interest are: 1) a gene involved in nitrogen metabolism that is strongly stimulated by phenylacetic acid and may have an impact in penicillin biosynthesis, and 2) a gene involved in glutathione reduction that may affect the levels of ACV/bis-ACV. The *P. chrysogenum* NCPC10086 genome carries 8 copies of the *pen* gene cluster and has several synonymous and non-synonymous mutations in the coding and non-coding sequences of the *pen* gene cluster. The improvement program of *P. chrysogenum* NCPC10086 produced two large translocations as compared to *P. chrysogenum* Wisconsin 54-1255. One of them include a 266 kb DNA fragment, encoding 107 genes [45]. The translocated fragment includes the nitrogen regulation gene *nre* and therefore may affect nitrogen metabolism in *P. chrysogenum* NCPC10086. A second large translocation has resulted in the transfer of a 1202 kb fragment; this fragment contains an ATP/ADP mitochondrial carrier protein that is involved in energy metabolism and also the *pex2* gene that encodes a peroxin protein involved in the peroxisomes structure [45].

The draft genome of two different *P. chrysogenum* strains, *P. chrysogenum* KF-25 and *P. chrysogenum* strain HKF2 have been published [49,50], although it is unknown whether these strains belong to the *P. rubens* clade. The first one was isolated from a soil sample in China and investigated for its ability to form antifungal proteins. The second one was isolated from a sludge treatment plant in India, and has interest for its ability to convert carbohydrates useful for the production of prebiotics. However, no detailed analysis of the penicillin or other secondary metabolites gene clusters has been made in these strains.

## 3. Transcriptomics

Discovery of bacterial enzymes enable specific cutting of the DNA molecules by means of restriction sites promoting the whole genomes mapping in early 1970s [51]. Subsequently, the DNA transfer and probing technique described by Edward Southern in 1975 allowed detection one single gene among thousands of DNA fragments. It was the so-called Southern blotting [52]. A couple of years later, Alwine and colleagues mimicked the DNA blotting system to transfer RNA molecules to a chemically activated cellulose paper [53]. The pun on Southern’s name generated the “Northern blotting” designation. A few years later, nomenclature was completed when proteins were (electro)blotted to nitrocellulose membranes and the methodology was dubbed “Western blot” [54,55,56]. Regarding the transcriptome analyses, the revolution began with the microarrays developed by Brown and DeRisi in the late 1990s, which allowed the analysis of thousands of genes in a single experiment [57,58]. Initially, it involved the hybridization of target mRNA transcripts to specific DNA probes but later the mRNA hybridization was against oligonucleotides [59]. Presently, microarrays have been displaced by the high-throughput technique of RNA sequencing (RNA-seq), which was established by Nagalakshmi and co-workers [60].

Transcriptome-wide analyses of *P. chrysogenum* began with the publication of the genome of this filamentous fungus [36]. Several studies have been conducted to characterize the transcriptional response of several *P. chrysogenum* strains under different scenarios. These are some examples.

### 3.1. Transcriptomics and Strain Improvement

Together with the analysis of the genome sequence, van den Berg et al. [36] prepared proprietary DNA microarrays (Affymetrix) and used them to characterize the molecular basis of the improved penicillin productivity achieved during classical strain improvement programs. With this purpose, the transcriptomes of the sequenced strain (Wisconsin 54-1255) and the penicillin-G high-producing strain DS17690, which were grown in the presence and absence of the side-chain precursor phenylacetic acid in aerobic, glucose-limited chemostat cultures, were compared. Results indicated that transcription of the penicillin-G biosynthesis genes *pcbAB*, *pcbC*, *penDE*, and *phl* was higher in the high-producing strain. Similar behavior was observed with the genes involved in the biosynthesis of precursor amino acids for the biosynthesis of penicillin (valine, cysteine and α-aminoadipic acid) and with several genes encoding microbody proteins.

Some years later, the transcriptome analysis of wild-type and overproducing strains from two unrelated fungal β-lactam producers (*A. chrysogenum* and *P. chrysogenum*) was carried out [61]. In this work, *P. chrysogenum* NRRL 1951 (wild-type) and P2niaD18 (production strain niaD-) were grown in the rich Complete Culture Medium at 27 °C and 120 rpm for three days. Transcriptional analysis from biological duplicates of each strain was carried out using RNA-seq with single end sequencing (read length 50 nt) being performed on the Illumina HiSeq 2500 platform. Data revealed that 748 genes (6.7% of the annotated protein-coding genes) showed differential expression in P2niaD18 regarding the wild-type strain. Among these genes, 357 (47.7%) showed an increased transcription rate, whereas 391 (52.3%) exhibited decreased expression profiles. The authors then performed a functional categorization of those genes with FungiFun2 [62] using the FunCat catalog of *P. chrysogenum* and *A. chrysogenum* [63]. The main findings include the up-regulation of genes from primary metabolism, mainly those involved in the supply of precursors for β-lactam biosynthesis. The penicillin gene cluster, which is duplicated in the penicillin high-producing strain, is more than four-fold up-regulated. Interestingly, the category “secondary metabolism” was present only in the set of down-regulated genes, with 27 of the 54 secondary metabolite gene clusters being differentially regulated in the industrial strain. For example, data from RNA-seq experiments led to the conclusion that pigment production is transcriptionally repressed in the improved strain. In this study, these authors also found that a large set of Velvet-regulated genes were differentially expressed during strain improvement (see below). Expression of cellular transport-associated genes and cell rescue, defense and virulence genes was down-regulated in the P2niaD18 strain [61]. These results were consistent with those reported in previous proteomics studies comparing wild-type and improved strains of *P. chrysogenum* [64,65].

### 3.2. Transcriptomics and the Metabolism of the Benzylpenicillin Side-Chain Precursor

As indicated above, van den Berg et al. (2008) characterized through microarrays (Affymetrix) the transcriptional response of the sequenced strain (Wisconsin 54-1255) and the penicillin-G high-producing strain DS17690 in the presence and absence of phenylacetic acid in aerobic, glucose-limited chemostat cultures [36]. Results indicated that transcription of the penicillin-G biosynthesis genes *pcbAB*, *pcbC*, *penDE*, and phl, although higher in the high-producing strain, was independent of phenylacetic acid. Interestingly, structural genes from the homogentisate pathway (involved in phenylacetic acid degradation) showed increased transcript levels in the presence of phenylacetic acid in both strains, despite the well-known inactivation of this catabolic pathway in the Wisconsin54-1255 strain and, presumably, in derived strains as well. The presence of phenylacetic acid exerted a strong effect in the up-regulation of gene expression from the functional categories of metabolism, transport, and detoxification [36].

Shortly after the publication of the *P. chrysogenum* genome, Harris et al. [66] characterized the genome-wide gene expression responses of *P. chrysogenum* to phenylacetic acid consumption and penicillin-G production employing DNA microarrays (Affymetrix). To achieve this goal, these authors engineered a strain (DS50661) without the penicillin gene cluster (*pcbAB*-*pcbC*-*penDE*), which was derived from the high-producing strain DS17690. The parental and modified strains were cultured under aerobic glucose-limited chemostat conditions in the presence and absence of phenylacetic acid. Interestingly, the penicillin biosynthetic genes were not differentially expressed under these conditions in the high-producing strain. On the other hand, the addition of the benzylpenicillin side-chain precursor gave rise in both strains to a strong up-regulation of the homogentisate pathway for phenylacetic acid catabolism, together with an increase in the expression of the gene encoding the phenylacetyl-CoA ligase (*phl*, Pc22g14900) and in the transcription rate of several genes in the functional categories “cellular transport and transport mechanisms’ and ‘transport facilitation”. Culture under penicillin-G production conditions led to transcriptional up-regulation of several genes involved in nitrogen and sulfur metabolism. Regarding the effect of the absence of the penicillin gene cluster on gene expression, this resulted in transcriptional up-regulation of a gene cluster, which included the paralog of the aristolochene synthase gene (*Ari1*) from *Penicillium roqueforti*, and therefore might be involved in production of the secondary metabolite aristolochene and its derivatives [66]. This gene cluster has been shown to encode the enzymes responsible for the biosynthesis of PR toxin [67].

Another interesting study related to the metabolism of phenylacetic was performed by Veiga et al. [68]. These authors confirmed that the side-chain precursor phenylacetate can be formed from phenylalanine, which undergoes transamination to phenylpyruvate and further conversion by decarboxylation or oxidation and hydroxylation to tyrosine, the latter being subsequently metabolized via the homogentisate pathway. In this work, possible pathways for phenylalanine metabolism in *P. chrysogenum* were investigated through a comparative transcriptome analysis using DNA microarrays (Affymetrix) of aerobic glucose-limited chemostat cultures of *P. chrysogenum* DS17690 (high-producing strain) grown with either phenylalanine or ammonium sulfate as the nitrogen source. A total of 331 genes were differentially expressed between both culture conditions (291 genes were overexpressed in the phenylalanine cultures and 40 genes were overexpressed in the presence of ammonium sulfate). Culture in phenylalanine led to the overrepresentation of transcripts from genes involved in the degradation of aromatic, branched-chain, and glutamate-, proline-, and sulfur-containing amino acids. Moreover, some of the 291 genes that were found overexpressed in the presence of phenylalanine encoded proteins that were expected to belong, based on sequence homology, to the homogentisate pathway. Some of them had been previously shown to be overexpressed in chemostat cultures supplemented with phenylacetate [66]. These results together confirmed the involvement of the homogentisate pathway in phenylalanine catabolism in *P. chrysogenum* [68]. Another group of genes showing overexpression in phenylalanine cultures encoded uncharacterized hydroxylases, known transaminases, two putative aldehyde dehydrogenases, and two putative phenylpyruvate decarboxylases. The introduction of one of these putative phenylpyruvate decarboxylase genes (Pc13g09300) in a *Saccharomyces cerevisiae* strain deficient in decarboxylase activity (deleted in the five decarboxylase genes encoding pyruvate decarboxylase isozymes, phenylpyruvate decarboxylase with a broad substrate specificity and a putative decarboxylase) restored the growth of this yeast mutant on glucose and phenylalanine, which confirmed that Pc13g09300 encodes a dual-substrate pyruvate and phenylpyruvate decarboxylase playing a key role in an Ehrlich-type pathway for the production of phenylacetate in *P. chrysogenum* [68].

### 3.3. Transcriptomics and the Impact of the Velvet Regulatory Complex

The first report about the transcriptional characterization of the impact of the Velvet regulatory complex on penicillin biosynthesis was carried out by Hoff and co-workers [31]. By using the custom-designed Affymetrix GeneChip^®^ DSM_PENa520255F (representing the genome of *P. chrysogenum* Wisconsin54-1255 [36]), a comparative transcriptome-wide analysis was carried out between the ΔPcvelA strain (a PcvelA knock-out mutant of the high-producing strain *P. chrysogenum* P2niaD18) and its parental strain, which were grown on solid medium for 48, 60, and 96 h. The study showed that knocking out of the PcvelA gene resulted in the down-regulation of the expression of 858 genes (6.6% of all nuclear genome) and up-regulation of 913 genes (7% of the nuclear genome), respectively, at least one of the three time points. Most genes exhibited differential expression after 96 h of growth. A subset of 154 sequences showed lower expression levels across all time-points, whereas 47 genes can be found in the common subset of up-regulated sequences at the three time-points. Results indicated that the expression of the Pc*laeA* gene was up-regulated in the ΔPcvelA strain. Data suggested that PcVelA acts both as an activator and a repressor of secondary metabolism. Regarding penicillin biosynthesis, the three biosynthetic genes *pcbAB*, *pcbC*, and *penDE* were strongly down-regulated in the ΔPcvelA strain, which demonstrated the regulatory role of PcVelA on penicillin biosynthesis [31].

The effect of Pc*velA* and Pc*laeA* deletions was also studied in aerobic, glucose-limited chemostat cultures of *P. chrysogenum* DS17690, another penicillin high-producing strain [47]. For this purpose, knock-out mutants in PcLaeA (PclaeAΔ) and PcVelA (PcvelAΔ) were constructed from the DS17690 strain and subjected to full transcriptome analysis using DNA microarrays (Affymetrix). Differential expression in both deleted mutants regarding the parental strain can be summarized as follows. In the absence of phenylacetic acid, 9 genes were up-regulated, whereas 23 genes were down-regulated. On the other hand, the lack of benzylpenicillin side-chain precursor led to the up-regulation of three genes and down-regulation of two genes. The group of 23 genes was the only one with a consistent differential response and included 11 genes, which belonged to two small gene clusters, one of which contained a gene with high homology to the aristolochene synthase (involved in the synthesis of toxins of sesquiterpenoid nature). Interestingly, neither PclaeAΔ nor PcvelAΔ showed drastic transcriptional changes in any of the eight Velvet complex orthologs (with the obvious exception of the deleted genes). It was also observed that the expression of the penicillin biosynthetic genes was not strongly affected in the deleted strains [47]. This was in contrast with previous studies reporting a key role of Pc*velA* and/or Pc*laeA* in the expression of the *pcbAB*, *pcbC*, and *penDE* genes in different strains of *P. chrysogenum* [28,31], a discrepancy that was explained in the way that the role of the Velvet complex can be strongly dependent on the context (i.e., prolonged batch culture or aerobic glucose-limited culture) [47].

**Figure 3:**
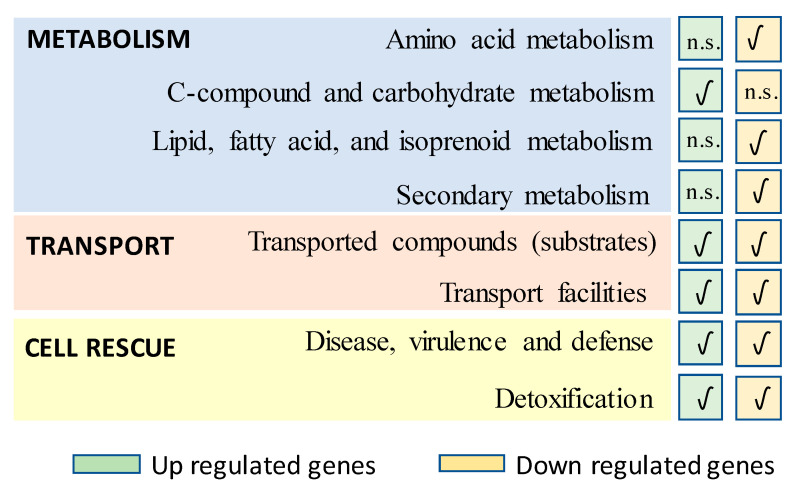
Enriched functional categories (FunCat) of differentially expressed genes after the transcriptome analysis between ΔPcvelA and the parental strain P2NiaD18. Significantly enriched categories either for up or down-regulated genes are represented by a check mark icon, whereas n.s. indicates non-significant enrichment. Adapted from [61].

As indicated above, Terfehr and co-workers in 2017 [61] carried out the transcriptome analysis of wild-type and overproducing strains from two unrelated fungal β-lactam producers (*A. chrysogenum* and *P. chrysogenum*), and found that Velvet-regulated genes are major targets during strain improvement. A ΔPcvelA strain was used to characterize Velvet-dependent transcriptional changes [69]. High-throughput RNA sequencing identified 567 differentially regulated genes (248 up-regulated and 319 down-regulated), which represents 5% of the annotated genes. Differentially expressed genes were assigned to functional categories based on the FunCat database and used for subsequent enrichment analysis. In the set of up-regulated genes an enrichment of the categories “C-compound and carbohydrate metabolism”, “transported compounds (substrates)” and “transport facilities”, and of the second-level categories “disease, virulence and defense” and “detoxification” was observed. On the other hand, overrepresentation of the categories “lipid, fatty acid, and isoprenoid metabolism”, “transported compounds (substrates)”, “transport facilities” and “disease, virulence and defense”, and of the second-level categories “secondary metabolism”, “detoxification” and “amino acid metabolism” was remarkable in the down-regulated gene set (Figure 3). Interestingly, 26.2% of 748 differentially expressed genes found during strain improvement were regulated in both the velvet mutant and production strain. Transcriptomics data from the ΔPcvelA strain indicated that the expression of about 50% of all secondary metabolite clusters, including β-lactam biosynthesis genes, is controlled by Velvet, with roughly half of the differentially expressed clusters being up- or down-regulated, thus suggesting that this regulator plays both a positive and a negative role in the expression of secondary metabolism gene clusters [61].

### 3.4. Transcriptomics under Environmental Stress Conditions

The phenomenon known as strain degeneration consists of a loss of production capacity upon long-term cultivation, such as chemostat cultivation, and is often observed during the production of non-catabolic products (e.g., antibiotics). Degeneration of penicillin production during prolonged ethanol-limited chemostat cultivations was tested in the high-producing strain *P. chrysogenum* DS17690 [69]. This strain was grown in the presence of 0.25 Cmol/L ethanol and 4 mM phenylacetic acid using chemostat cultivations, and transcriptome analysis was carried out to investigate whether the observed degeneration was related to changes in gene expression. For this purpose, RNA samples were obtained throughout the fermentation period for up to 500 h, which represented about 22 generations. Analysis of gene expression was performed using Affymetrix custom-made *P. chrysogenum* GeneChip^®^ microarrays (DSM_PENa520255F). Interestingly, not significant down-regulation of the expression of the penicillin biosynthesis genes or of the genes encoding the global regulators PcVelA (Pc13g13200) and PcLaeA (Pc16g14010) was observed during degeneration. On the other hand, about 1000 genes showed significant differential expression throughout the prolonged chemostat cultures. The categories “nitrogen and sulfur metabolism” and “nitrogen and sulfur use” genes were down-regulated throughout the prolonged chemostat cultivations. Also, many of the down-regulated genes were related to the biosynthetic pathway of the penicillin-precursor amino acid cysteine [69].

Taking into account that *Penicillium* species are main contaminants in International Space Station and their enzymatic machinery is capable of degrading spacecraft hardware [70], a high-throughput de novo RNA sequencing study was conducted in *P. chrysogenum* grown under microgravity and normal gravity conditions to elucidate novel responses associated with microgravity [71]. In this study, *P. chrysogenum* KACC 425,892 was grown in a high aspect ratio vessel (HARV) designed by NASA to provide microgravity condition and the system was operated in a controlled chamber set at 25 °C and 90% humidity, thus avoiding the formation of bubbles. RNA samples were obtained at 12 and 48 h under microgravity and normal gravity conditions and sequenced on an Illumina Nextseq platform. At both time-points, an increase in the expression of the penicillin biosynthetic *penDE* gene (encoding IPN acyltransferase) was observed under microgravity conditions. Besides the effect on carbohydrate metabolism and secondary metabolism (including penicillin biosynthesis), a slight up-regulation of the genes involved in pathogenicity, together with an increased response to environmental stimulus and stress tolerance, were also reported under microgravity conditions. In addition, it is worth mentioning that microgravity led to the overexpression of some ABC and MFS transporters [71].

### 3.5. Transcriptomics and Production of Cephem Antibiotics by Engineered Strains of P. chrysogenum

Engineering of *P. chrysogenum* for the production of cephem compounds is interesting from the industrial point of view, and the production of semisynthetic cephalosporins using this approach requires supplementation of the growth media with the side-chain precursor adipic acid.

In one study, Harris and co-workers genetically modified the *P.chrysogenum* penicillin-G high-producing strain DS17690 (introduction of the expandase-hydroxylase *cefEF* gene from *A. chrysogenum* and the 3´-hydroxymethylcephem O-carbamoyltransferase *cmcH* gene of *S. clavuligerus*) for the production of the important semisynthetic carbamoylated cephalosporin precursor adipoyl-7-amino-3-carbamoyloxymethyl-3-cephem-4-carboxylicacid (ad7-ACCCA), thus giving rise to the DS49834 strain [66,72]. The physiological impact of this modification was characterized using microarray-based (Affymetrix GeneChip^®^) transcriptomics studies performed in batch and aerobic glucose-limited chemostat cultures in the absence and presence of the side-chain precursor adipic acid. A congenic strain (DS50661), which lacks the benzylpenicillin biosynthetic gene cluster [66], was cultured in parallel under the same conditions to dissect the transcriptional responses to adipic acid per se and to ad7-ACCCA production. Comparative analyses between strains and conditions revealed a total of 288 transcripts (2% of the genome) being differentially expressed in at least one of the comparisons. Data revealed that the β-lactam biosynthetic genes were overexpressed at high levels in the engineered strain, regardless of the addition of adipic acid. A similar result was observed with the transcription of the heterologous genes *cefEF* and *cmcH*. However, adipic acid led to the overexpression of Pc22g20270, which would be later characterized as the *aclA* gene encoding a broad-substrate-specificityacyl-coenzyme A ligase involved in activation of adipic acid [73]. Analysis of gene expression also revealed that those genes participating in the catabolism of adipic acid via β-oxidation were overrepresented. Interestingly, under ad7-ACCCA conditions, overexpression of several genes related to secondary metabolism, detoxification, and transport were observed, together with the induction of four genes with homology to glutathione S-transferases, thus pointing to the oxidative stress response as an adaptive mechanism to the production of ad7-ACCCA [72].

Another study focused on the effect of adipic acid upon the production of cephem compounds in engineered *P. chrysogenum* strains was conducted by Veiga and co-workers [74]. Chemostat cultures of *P. chrysogenum* DS17690 (penicillin high-producing strain) [73], DS49834 (a strain producing the cephalosporin precursor ad-7-ACCCA) [73] and DS50661, a strain lacking the three penicillin biosynthetic genes [66] were conducted in the presence and absence of adipic acid, and transcriptional analysis was performed through DNA microarrays (Affymetrix). This experiment confirmed a previous study reporting that adipic acid metabolism in this fungus occurs via β-oxidation [72], since within the group of 52 genes transcriptionally up-regulated in the presence of adipic acid in these strains, peroxisomal genes involved in β-oxidation were enriched [74]. They included six genes encoding putative acyl-CoA oxidases and dehydrogenases, which are responsible for the first step of β-oxidation. In fact, deletion of the genes encoding the acyl-CoA oxidase Pc20g01800 and the acyl-CoA dehydrogenase Pc20g07920, improved the biosynthesis of the semisynthetic cephalosporin intermediate adipoyl-6-APA and reduced the consumption of adipic acid, which suggested potential targets of the first step of β-oxidation towards the improvement of cephalosporin biosynthesis [74].

## 4. Proteomics

In 2011, a century after Sir Joseph built the first instrument able to measure mass-to-charge (*m/z*) values of gaseous ionized atoms [75], McLafferty, on the one hand, and Yates, on the other hand, described how the mass spectrometry reached the Molecular Biology laboratory [76,77]. It was on 90s when spectrometry was finally applied to Biology and especially in Proteomics. This fact was supported by the informatics development, which allows equipment size decrease, higher mass resolutions, faster data acquisition, and generation of massive genes and genomes databases, which really allowed the biological applications [77]. The description of the ‘rapid rise of Proteomics’ in the post-genome era in a manuscript published by Peter James in 1997 was the starting point of the term Proteomics [78]. Initially, Proteomics findings were based on bidimensional gel electrophoresis even though it is no longer the only experimental approach used in modern Proteomics [79]. Thus, the combination of the sodium dodecyl sulfate (SDS) protein electrophoresis as described Laemmli [80] with the denaturing isoelectric focusing defined by O’Farrell [79,81] allowed to the bidimensional separation of proteins combining the isoelectric point and molecular weight. Up to date, this methodology has been the most widely used Proteomics procedure in *P. chrysogenum*.

Proteome analysis of different strains of *P. chrysogenum* has given Proteomics the chance to enhance the understanding of the penicillin production physiology by means of a reverse engineering process as recently reviewed by Barreiro and García-Estrada [3]. Thus, data collected from the wild-type strain (*P. chrysogenum* NRRL 1951), the reference laboratory strain (*P. chrysogenum* Wisconsin 54-1255) and several high penicillin production strains (*P. chrysogenum* AS-P-78, *P. chrysogenum* DS17690; *P. chrysogenum* QMSHB-11-06) have boosted the proteome analyses and knowledge of penicillin production [64,65,82,83].

The proteome is a growth phase-, external stimuli-, cell function-, or location-dependent variable entity, which contrasts with the theoretical static behavior of the genome [84,85]. In summary, the proteome analysis compares snapshots of punctual situations, which allows us to infer cellular stages. These snapshots can be used for different purposes and the simplest classification divide their analyses in three categories [86]. First, the expression proteomics that aims to study the qualitative and quantitative expression of total genes under different conditions. Second, the structural proteomics or cellular map proteomics, which provides detailed information about protein complexes present in a specific cellular organelle or system (e.g.: membrane, ribosomes, cell organelles). Finally, the functional proteomics that provides information about protein functions through the identification of interacting protein partners. Expression proteomics analyses have been carried out in penicillin-producer strains to compare between different them or between different culture conditions [64,65,83,87,88,89,90]. However, structural proteomics have been used to achieve reference maps of different sub-proteomes (e.g.: microbodies, cytoplasmic and secretome) [64,65,82].

### 4.1. Reference Maps of Different P. chrysogenum Sub-Proteomes

Reference maps are a methodological proof of concept or tour de force of the technological approach used. Moreover, they allow updating the sampling, extraction, and analytical procedures, which guarantee a later successful proteome analysis. Barreiro and co-workers [91] summarized the upgrading of several procedures adapted or specifically developed for *P. chrysogenum* and other filamentous fungi. Even though the Proteomics basic steps are well known, every microorganisms or growth conditions need a specific fine-tuning [92]. Furthermore, proteomics data can improve genome annotations and to discover transcript variants and protein isoforms due to alternative splicing detected through the detected proteins [93,94].

The penicillin biosynthesis is compartmentalized between cytosol and microbodies [95]. This fact justifies that the first reference maps of *P. chrysogenum* were the microbodies [82] and the intracellular [65] sub-proteomes maps, respectively. In 2008, the genome sequence of *P. chrysogenum* Wisconsin 54–1255 was published [36]. Interestingly, it was observed that microbodies (peroxisomes) are more abundant in high-producer strains than in wild types. Moreover, the transcription of genes encoding microbody proteins is enlarged in penicillin high-producing strains, which focused the attention on these organelles [36]. Thus, Kiel and co-workers [82] reported in 2009 the proteome of the microbody matrix of a *P. chrysogenum* high penicillin-producing strain (DS17690). Initially, an *in silico* analysis resulted in 200 predicted proteins containing a peroxisomal targeting signal (PTS), where 190 were putative proteins with PTS1 and 10 were putative PTS2 proteins. Subsequently, the microbody fractions of *P. chrysogenum* was purified and microbody matrix protein fractions were resolved by SDS-PAGE minigels (7.5% or 12.5%), tryptically digested and finally analyzed by LC-MS/MS in a MALDI-TOF/TOF instrument. As a result, 89 putative microbody proteins were identified by mass spectrometry, 79 contained a PTS and 10 lacked it. From those, 69 out of the 79 PTS-containing proteins experimentally identified were previously defined in the reference list. The most relevant identified components related to penicillin production were the last penicillin biosynthetic enzyme IAT (IPN acyltransferase; Pc21g21370), the isocitrate lyase (ICL1, Pc22g03660) and a novel fumarate reductase-cytochrome b5 fusion protein (Pc12g03090).

Immediately after the genome of *P. chrysogenum* was published in 2008 [36] and the microbodies proteome analyzed in 2009 [82], two additional reference maps were performed by Jami and co-workers in 2010, the intra-cellular proteome [65] and the secretome [64]. Due to the location of the penicillin biosynthetic enzymes, the intra-cellular proteome is highly relevant for the process. Thus, by means of bidimensional electrophoresis (2-DE) using a wide pI range (pH 3–10 NL) and 15% SDS-PAGE, up to 976 spots were detected, which yield 549 different identified proteins by peptide mass fingerprinting (PMF) and tandem mass spectrometry analysis (MALDI-TOF/TOF). It represents 4.24% of the 12,943 predicted nuclear-encoded proteins of the *P. chrysogenum* genome. The main group of identified proteins (43.6%) belongs to the Energy and Metabolisms class. Two of the penicillin biosynthetic enzymes (IPN synthase and IAT) were located in the reference map. However, the first enzyme in the pathway (ACV synthetase) due to its large molecular mass (425 kDa) was not observed. Interestingly, the largest represented proteins were two isoforms of a probable flavohemoglobin Fhp (Pc12g14620), which constituted 4.63% of the total relative protein quantified volume. Moreover, this flavohemoglobin was identified in 14 different isoforms [65].

**Figure 4:**
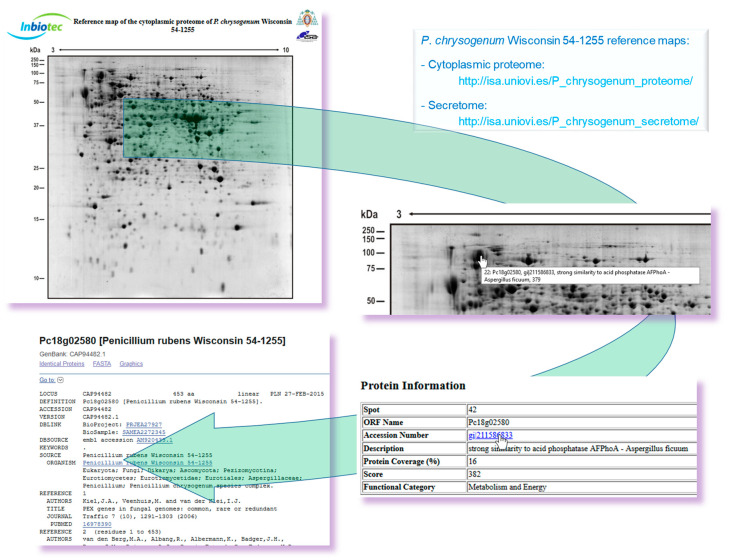
Websites of the intra- and extra-cellular proteome reference maps of *P. chrysogenum* Wisconsin 54-1255. The consecutive views obtained by clicking over each identified protein spot are presented.

Enzyme cocktails secreted by fungi have remarkable use across several industrial processes, which is well-known even more than a century after the first biotechnology patent, as Baker described in 2018 [96]. Filamentous fungi have an extraordinary ability to secrete secondary metabolites, organic acids, as well as proteins to the culture medium, which plays relevant roles in their sustenance, colonization, and pathogenicity. This secretory capacity made filamentous fungi attractive platforms for industrial production of extracellular proteins (e.g., food, beverages, plant biomass degradation) [64,97,98]. However, the first concern to analyze the extracellular proteome or secretome is to guarantee the proteins quality to avoid the analysis of those resulting from the lysis process (degradome) [6]. Thus, the initial step of the *P. chrysogenum* Wisconsin 54-1255 secretome analysis was the sampling point definition based on the growth phases over time (24 h, 40 h, 68 h, 74 h, and 86 h). When the growth (dry weight) was compared to the protein secretion (Bradford quantitation) in *P. chrysogenum*, it was observed that time-points later than 68 h correspond to cell lysis events, which could interfere with the secretome analysis [64]. A 2-DE (bidimensional electrophoresis) approach though a pH range from 4 to 7 and a 12.5% SDS-PAGE yielded 279 spots, which represent 131 different proteins (62 proteins showed a total of 259 isoforms and 69 proteins had no isoforms). Predictive software’s were used to analyze their possible classical (SignalP) [99] or non-classical (SecretomeP) [100] signal sequences present in the identified proteins, which yield 102 positives out of these 131 differentially identified proteins in the extracellular protein fraction. The original *P. chrysogenum* strain was initially isolated from a cantaloupe [101], which strongly support the large number of extracellular proteins related to its plant saprophytic behavior. Moreover, several IgE-binding proteins and antigens were also identified, which highlights the significance of *P. chrysogenum* as an indoor allergen [64]. The reference maps of the cytoplasmic proteome and secretome are available on-line (http://isa.uniovi.es/P_chrysogenum_proteome/ and http://isa.uniovi.es/P_chrysogenum_secretome/, respectively) (Figure 4).

### 4.2. Expression Proteomics Analyses

Once penicillin was discovered, one of the problems that researchers faced up was the low penicillin titers of the Fleming’s isolated strain, which led scientists to look for more productive microorganisms. On the one hand, new penicillin producer strains were isolated, such as *P. chrysogenum* NRRL 1951 (isolated as a saprophyte from a moldy cantaloupe); and on the other hand, higher productivity yields were reached through mutagenic treatments (e.g.: UV light, X-ray irradiation). As a consequence of these strain improvement programs, impressive penicillin titers were reached (at least three orders of magnitude) [6], thus giving rise to several industrial strains that are available, presently. These strains constitute a perfect evolution tracking system of the penicillin industrial improvement process of *P. chrysogenum*.

INBIOTEC’s researchers selected three strains to analyze the evolution of the penicillin production process by means of the intra- [65] and extra-cellular [64] proteomes. As a control, the wild-type strain *P. chrysogenum* NRRL 1951 (low penicillin titer) was selected. Secondly, as a moderately improved penicillin-producer, *P. chrysogenum* Wisconsin 54–1255 (reference laboratory strain) was used. Finally, a strain developed by the company Antibióticos SA (Spain), *P. chrysogenum* AS-P-78, was selected as an industrial type.

The intracellular proteins were analyzed by 2-DE with a pI wide range (pH 3–10 NL) and 12.5% SDS-PAGE. Initially, a reduction of proteins involved in the catabolism of penicillin precursors, such as cysteine and valine, as well as proteins involved in the biosynthesis of other secondary metabolites was observed in the Wisconsin 54-1255 strain compared to *P. chrysogenum* NRRL 1951. Consequently, an overproduction of proteins involved in valine and cysteine synthesis was detected in the AS-P-78 strain. Due to the industrial culture conditions, several proteins related to host infection (plant-infecting mechanisms) and virulence were under-represented in the Wisconsin 54-1255 strain. These genes are not needed when the nutrients are industrially supplied in big fermenters. Moreover, these virulence proteins were even less represented in the industrial strain AS-P-78.

It is well-known that an increase in the biosynthetic genes, a decrease of the final product degradation proteins, elimination of non-relevant nutrient drains, and a better antibiotic precursor supply are key steps to enhance the microbial secondary metabolites production [102]. This is the reason the energy supply (ATP) and redox balance need to be redistributed too. Thus, some proteins related to glycolysis and energy formation were detected as overrepresented in the Wisconsin 54-1255 strain, compared to the *P. chrysogenum* NRRL 1951 strain. Moreover, several proteins involved in redox metabolism were detected as predominant in the wild-type strain (NRRL 1951). Intriguingly, the high-production strain (AS-P-78) showed an increased flux through the pentose phosphate pathway and a decreased expression of some glycolysis proteins. A remarkable finding was the overproduction of proteins involved in oxidative stress response in the AS-P-78 strain, which suggests an industrial conditioning process. These facts indicate a metabolic realignment to increase the penicillin titers.

The secretome (extracellular proteome) was analyzed by using bidimensional electrophoresis in a 12.5% SDS-PAGE and a 4-to-7 pH range [64]. As previously indicated, *P. chrysogenum* has a saprophytic way of life. Thus, it secretome presented several proteins functionally involved in plant saprophytic growth. The comparison among the three strains (NRRL 1951, Wisconsin 54–1255 and AS-P-78) revealed that secretion of enzymes related to plant pathogenesis, infectivity, and tissue invasion were weakened in strains obtained by improvement programs. In contrast, the pectinolytic enzymes and proteases produced by the AS-P-78 strain was higher than in other strains, which suggests a role as white biotechnology cell-factories for these strains.

In parallel to an increase in penicillin titers due to the screening of new penicillin-producer strains, the production processes were also focused on deep-tank fermentation and nutrient supply [3,103]. The proteome analysis of the changes between pilot and industrial fermentation scales of *P. chrysogenum* was the fourth expression proteomics published [83]. The strain QMSHB-11-06 of *P. chrysogenum* grown in 50-L fermenters (pilot scale) and 150,000-L fermenters (industrial scale) was used to analyze its proteomes. A time course from 2 to 174 h was done and studied by 2D-PAGE and pH range of 4–7 combined with MALDI-TOF/TOF identifications. Several modifications were observed in different metabolic pathways, including the carbohydrate metabolic process, redox metabolism, amino acid, and nucleoside metabolic processes and penicillin biosynthesis. These results suggest that the industrial process might provide a friendly environment for amino acid metabolism to enhance the penicillin-precursor production (valine, cysteine, and α-aminoadipic acid). Furthermore, Cheng and co-workers [83] found an overproduction of the IAT and the CoA ligase during the industrial fermentation, which supports the higher penicillin production at industrial scale.

Regulation of the secondary metabolites production is a well-known microbial control system from bacteria [102,104] to fungi [105], but not fully unveiled. Thus, the fungal development and the secondary metabolism are connected and often coordinated in ascomycetous fungi through cellular signaling processes (e.g., sporulation) [106]. A traditional fungal global regulator is the heterotrimeric Gα protein Pga1, which is involved in conidiation, sporulation and penicillin biosynthesis [107,108,109,110]. Carrasco-Navarro and co-workers in 2016 [111] analyzed several *P. chrysogenum* Wisconsin 54-1255 mutants with diverse levels of activity of the Pga1-mediated signaling pathway by 2D-DIGE [112,113] (pH 3–10 range and 12% acrylamide SDS-PAGE). The analysis showed how the protein Pga1 is involved in ATP and NADPH control and cysteine biosynthesis, which are required at high levels for penicillin production. Thus, a subsequent in silico interactions analysis showed that the protein Pga1 is also involved in morphogenesis, vegetative growth, stress response and penicillin biosynthesis [111].

Proteomics applied to *P. chrysogenum* has also analyzed the effect of several putative penicillin enhancer compounds, such as: phosphopeptides and CaCl_2_ [89]; phenylacetic acid [87] or 1,3-diaminopropane and spermidine [90]. Therefore, it has been described the enhancer effect of some polyamines (e.g.: 1,3-diaminopropane, spermidine) over the penicillin production, which contrasts with the lack of effect of other amines such as putrescine or spermine [114]. This effect is related to the expression of the penicillin biosynthetic genes *pcbAB*, *pcbC* and *penDE*, and appeared to be partially mediated by the global regulatory protein LaeA [115]. A comparative Proteomics analysis (pH 3–10 gradient followed by 12.5% SDS-PAGE) was carried out in *P. chrysogenum* Wisconsin 54-1255 cultures supplied with 1,3-diaminopropane or spermidine, which presented similar protein profiles [90]. This interconnection between these two molecules was supported by the overproduction of the spermidine synthase protein after 1,3-diaminopropane addition. Moreover, the analyzed polyamides induced: (i) the synthesis of a new isoform of IAT (the last penicillin biosynthetic enzyme), (ii) the biosynthesis of β-alanine involved in the synthesis of essential prosthetic groups for ACV synthetase (the first enzyme of the penicillin biosynthetic pathway); (iii) sporulation and (iv) intracellular content of vesicles, which play an important role in the biosynthesis of other secondary metabolites (e.g.: aflatoxins) [90,116,117]. In contrast, the two studied polyamines reduce the phenylacetic acid (side-chain precursor for benzylpenicillin) degradation by homogentisate catabolic pathway, which increase its bioavailability for penicillin biosynthesis [90].

Phenylacetic acid is a weak acid, which acts as specific side-chain precursor of the benzylpenicillin biosynthesis [118]. However, its catabolic degradation happens through the homogentisate pathway [8,9,10,119]. *P. chrysogenum* seems to survive this toxic compound due to its adaptation mechanisms under benzylpenicillin production conditions [3,6]. This metabolic rearrangement to detoxify the phenylacetic acid is an intriguing concern faced up by proteome analysis. Thus, intracellular (pH 3–10 gradient) and extracellular (pH 4–7 gradient) proteomes were investigated by 2-DE (12.5% polyacrylamide SDS-PAGE) and subsequent protein identification by mass spectrometry in a MALDI-TOF/TOF instrument [87]. In the intracellular proteome, several enzymes related to β-lactam biosynthesis were overrepresented when phenylacetic acid was present: (i) the IAT protein (catalyzing last step of the penicillin biosynthetic pathway), (ii) proteins of cystathionine (precursor of penicillin-precursor cysteine) and L-valine (precursor of penicillin) biosynthesis, (iii) a probable thioredoxin peroxidase involved in the ACV reincorporation to the penicillin biosynthetic pathway, and (iv) the S-adenosylmethionine synthase, which coordinates the fungal secondary metabolism and development [120]. When the extracellular proteome was analyzed some proteins lacking a predicted signal sequences for secretion were observed, such as a NADPH-dependent glutamate dehydrogenase (encoded by the *gdhA* gene), an enzyme involved in the production of β-lactam antibiotics in industrial strains of *P. chrysogenum* [87,121].

The addition of calcium salts in the culture medium has a positive effect in penicillin production and it also plays an important role in the growth of *P.* chrysogenum, whereas casein phosphopeptides are able to chelate Ca^2+^ increasing the proteins secretion in *Aspergillus* strains [88,122,123]. The combination of both additives (Ca*^2+^* and casein phosphopeptides) increased 10–12-fold penicillin production in *P. chrysogenum* as described Domínguez-Santos and co-workers in 2017 [89]. The two-dimensional-differential in gel electrophoresis (2D-DIGE) technology was used in *P. chrysogenum* in a 3–10 non-linear pH range and 12.5% polyacrylamide SDS-PAGE analysis. Proteome analysis demonstrates the effect of Ca*^2+^* plus casein phosphopeptides over penicillin through several ways. They directly act over (i) the biosynthetic pathway since two penicillin biosynthetic enzymes (IPN synthase and IAT) are overexpressed; (ii) the energy and NADH formation; (iii) the glycolysis and methylcitrate cycles, as well as, over (iv) some aminases and aminotransferases involved in glutamate formation, which induce the intracellular concentration of the ACV synthetase and penicillin biosynthesis [124]; and over (v) the biosynthesis of cystathione, which keep the appropriate cysteine pools [125]. Furthermore, a peroxisomal catalase, which mediates the high resistance to fungal oxidative stress present in penicillin high-producing strains [65], was found overproduced. Other peroxisome matrix proteins or microbody-associated proteins were observed as induced by Ca*^2+^* and phosphopeptides. This fact strongly suggests the effect of these compounds over the penicillin biosynthetic enzymes in peroxisomes and vesicles [89].

Presently, other *Penicillium* strains, apart from *P. chrysogenum*, are being analyzed under the Proteomics point of view. New methodologies approaches, such as the label-free protein profiling, are being used. Thus, it has been recently used to observe the effect of antifungal agents (e.g.: 2-methoxy-1,4-naphthoquinone) over the pathogenic strain *Penicillium italicum* or the action of co-cultured biocontrol agents (*P. chrysogenum*, *Debaryomyces hansenii*) with the toxigenic mold *Penicillium nordicum*, typically present in dry-cured meat products [126,127]. Omics combinations have also been used in *Penicillium* genus. For example, RNA-seq and iTRAQ were combined to understand the differentially expressed genes involved in spore germination of *Penicillium expansum* [128]. All these facts strongly suggest the wide scope able to be unveiled by means of Proteomics applied to the fungal strains.

## 5. Metabolomics

Metabolomics-wide analyses are focused on the study of the chemical fingerprints that cellular processes leave behind through the analysis of all metabolites in an organism, which is called metabolome [129]. Although data obtained from genomics, transcriptomics and proteomics comparative analyses reveal information about the set of gene products produced in the cells under different conditions, metabolomics approaches provide useful information about the physiology of the cells [130]. Nevertheless, monitoring metabolome changes is experimentally tedious and complex.

Metabolomic samples contain multiple metabolites that are usually first separated, and then identified and quantified. High performance liquid chromatography (HPLC) is the most common separation technique used in metabolomic analysis coupled with electrospray ionization tandem mass spectrometry (LC-ESI-MS/MS) used to identify and quantify metabolites after separation [131]. Although this analytical technique is powerful, it still has limitations to obtain an accurate quantitative metabolic profiling. These drawbacks are due to ion enhancement or ion suppression in the electrospray ionization caused by matrix effects and the degradation of metabolites during sample preparation [132]. Some of these problems have been overcame by the addition of isotopically enriched standards for each compound of interest to the analyzed sample. The Isotope-Dilution Mass Spectrometry (IDMS) has been proven to be the most reliable technique for the generation of accurate quantitative metabolite data [132,133].

The analysis of all metabolites in a biological system through comparative metabolomics approaches not only provide information about metabolite concentrations, but also these analyses allow the establishment of a link between metabolites and the pathways they are involved [134,135,136,137,138].

In *P. chrysogenum* sensing of nutrients and signal transduction takes place by complex regulatory cascades that control expression of penicillin and other secondary metabolites [139], thus connecting primary and secondary metabolism. As an example, a metabolome study shows the relation between the penicillin production flux and central metabolism in *P. chrysogenum* under different perturbation conditions affecting the penicillin pathway and central metabolism. In this study, a high penicillin-producing strain was cultivated in glucose-limited chemostat cultures under penicillin-G producing and non-producing conditions. The absence of penicillin production triggered significant changes in the flux through central metabolism leading to changes in concentration of central metabolites. A positive relation was observed between metabolite concentration and carbon flux in central metabolism, which means that a decrease in the flux through the tricarboxylic acid (TCA) cycle also resulted in a decrease in the intermediate metabolites involved. This positive relation was also observed with most of the free amino acids and their precursors in central metabolism [125]. Data from these experiments showed that the flux through penicillin production pathway was only influenced by cysteine and the ATP levels. Since synthesis of cysteine requires a large NADPH supply, it can be said as a final statement that penicillin production mostly depend on energy and redox status of the cells as previously was observed through the intracellular proteome analysis [65]. Another interesting finding was that the absence of penicillin production increased storage metabolism [125].

Although major improvements have been achieved in *P. chrysogenum* to increase penicillin productivity, there is still room for further improvement and metabolomics creates new possibilities. Metabolomics analysis clearly come out that penicillin production is highly influenced by central metabolism meaning that successful improvement in penicillin production should not be limited to metabolic engineering of the penicillin pathway alone, but be applied in concert with the parts of central metabolism involved.

## 6. Conclusions

**Figure 5:**
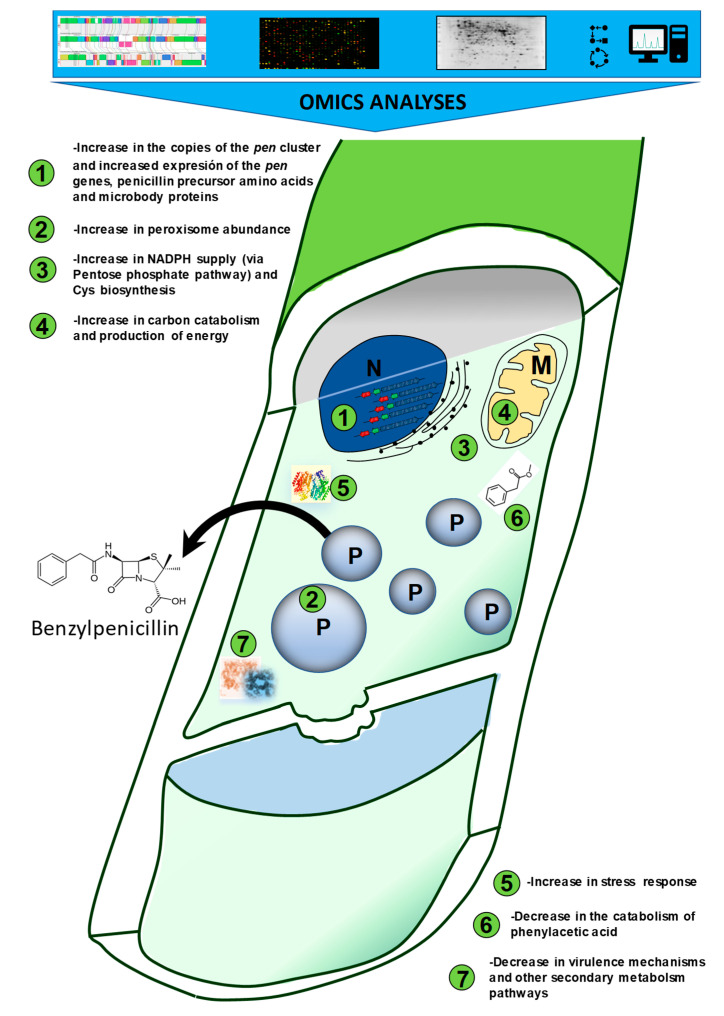
Summary with the main findings focused on penicillin production and provided by omics analyses showing the modifications undergone by *P. chrysogenum* throughout the strain improvement programs.

We are close to celebrating the 100th anniversary of the discovery of penicillin at the end of this decade. Although this antibiotic and the fungal microorganism producing it have been extensively characterized during these years, only the arrival of the post-genomic era at the beginning of the 21st century has shed light into the industrial secrets behind penicillin production. Early after the production of penicillin attracted the interest of industry, *P. chrysogenum* was subjected to strain improvement programs aimed to boost penicillin titers. This microorganism was able to adapt to the stress conditions of industrial production and only global omics analyses have provided the information about the deep metabolic reorganizations occurring during the improvement process (Figure 5).

All the information gathered about penicillin biosynthesis and metabolic reorganization of improved strains can be used to increase the production of current or new versions of penicillin in *Penicillium* or other producing microorganisms, and can be the model for new antibiotic producer strains. The need for new antimicrobial compounds, in addition to better production processes and improved microorganisms, can be based on Systems Biology and Synthetic Biology developments, which can benefit from the data generated by different omics.
